# Cancer functional states-based molecular subtypes of gastric cancer

**DOI:** 10.1186/s12967-023-03921-1

**Published:** 2023-02-04

**Authors:** Qi Zhou, Yiwu Yuan, Hao Lu, Xueqin Li, Ziyang Liu, Jinheng Gan, Zhenqi Yue, Jiping Wu, Jie Sheng, Lin Xin

**Affiliations:** 1 Department of General Surgery, The Second Affiliated Hospital of Nanchang University, No.1 Minde Road, Donghu District, Nanchang, 330006 Jiangxi China; 2 Jiangxi Province Key Laboratory of Molecular Medicine, The Second Affiliated Hospital of Nanchang University, Nanchang, Jiangxi China; 3 Nursing department, The Second Affiliated Hospital of Nanchang University, Nanchang, Jiangxi China

**Keywords:** Gastric cancer, Cancer functional states, Subtypes, Single cell

## Abstract

**Background:**

The treatment of gastric cancer remains a challenge.

**Methods:**

We divided gastric cancer into three subtypes based on 14 cancer functional states. We investigated differences between subtypes through multi-omics data, especially at the single-cell level, which allowed us to analyze differences from the perspective of each type of cell rather than the whole.

**Results:**

The cluster 1 is characterized by high levels of tumor progression-related cancer functional status, worst survival outcomes, low metabolic level, high infiltration of immunosuppressive cells, high copy number variations (CNV), and low tumor mutational burden (TMB). The cluster 2 is characterized by low levels of tumor progression-related cancer functional status, favorable prognosis, moderate metabolic level, low immune cell infiltration, high CNV, and moderate TMB. Then, the cluster 3 is characterized by the high level of all cancer functional status, high metabolic level, low CNV, high TMB, high infiltration of immune cells with high cytotoxicity, and better response to immunotherapy. We also established a prognostic model based on cancer functional status and validated its robustness.

**Conclusions:**

Collectively, our study identified gastric cancer subtypes and provided new insights into the clinical treatment of gastric cancer.

**Supplementary Information:**

The online version contains supplementary material available at 10.1186/s12967-023-03921-1.

## Introduction

Gastric cancer (GC) is the sixth most common cancer and the third leading cause of cancer-related deaths worldwide [1]. And the main endemic area of GC is East Asia [2]. There are various treatment methods for GC, including surgery, chemotherapy, radiation therapy, and targeted therapy and immunotherapy. With the progress of these treatment methods, the mortality rate of GC has decreased significantly in recent decades, but it is still a great social burden [3]. Gastric cancer, like other tumors, is a heterogeneous disease. The most commonly used clinical classification methods are Lauren classification, histological classification and American Joint Committee on Cancer (AJCC) staging system. There are also many researchers to classify GC patients based on the gene expression characteristics of GC samples, and The Cancer Genome Atlas (TCGA) molecular subtypes [4] and the Asian Cancer Research Group (ACRG) molecular subtypes [5] are more commonly used. A better understanding of the heterogeneity of GC will allow us to carry out more precise treatment of GC patients.

In recent years, immunotherapy has attracted widespread attention, and multiple immune checkpoint inhibitors (ICIs) have been used in the treatment of multiple malignant tumors [6, 7]. Despite huge advances in immunotherapy, only a subset of GC patients treated with ICIs show good responses to immunotherapy. Therefore, it is critical to identify which patients have response to immunotherapy.

So far, there are many studies on the classification of GC, but many of them are based on certain types of characteristics of GC [8–10]. In our study, we used 14 malignancy features to perform unsupervised clustering analysis of GC samples. These cancer functional states gene sets were obtained from an online analysis website (CancerSEA; https://biocc.hrbmu.edu.cn/CancerSEA/home.jsp) [11], including angiogenesis, apoptosis, cell cycle, differentiation, DNA damage, DNA repair, EMT, hypoxia, inflammation, invasion, metastasis, proliferation, quiescence and stemness. Based on the above cancer functional states, we divided GC into three subtypes. We conducted a detailed exploration on the characteristics of the three subtypes in terms of single nucleotide polymorphism (SNP), copy number variations (CNV), DNA methylation, proteomics, and tumor-infiltrating immune cells. Importantly, we also performed an exhaustive investigation of the differences between the various types of cells in the three subtypes at single-cell resolution. Overall, the classification of GC in our study may play a role in the precise treatment of patients.

## Materials and methods

### Data acquisition and processing

Gene expression profiles and clinical data for the TCGA-STAD cohort from The Cancer Genome Atlas (TCGA; https://portal.gdc.cancer.gov/) were downloaded using TCGAbiolinks package in R. The count value of TCGA-STAD cohort samples were converted into transcripts per million (TPM) value. Somatic mutation and copy number variations in TCGA-STAD cohort were downloaded using the package TCGAbiolinks in R. After we downloaded the CNV data from TCGA, we first calculated it using GISTIC2 and then visualized it using the maftools package [12]. Somatic mutation data were analysed using R package maftools [12].

Gene expression profiles and clinical data for GSE62254 [5], GSE15459 [13], GSE57303 [14], GSE34942 [15] and GSE84437 [16] were downloaded from the Gene-Expression Omnibus (GEO; https://www.ncbi.nlm.nih.gov/gds/). Because of the large number of datasets on the GPL570 platform, four datasets (GSE62254, GSE15459, GSE57303 and GSE34942) from the GPL570 platform were merged as one dataset named as GPL570 dataset using “oligo” package in R [17]. In the GPL570 dataset, we used the RMA method in the oligo package to normalize the expression values, and the combat method in “sva” package was used to remove batch effects [18]. The results obtained by the RMA method were log2 transformed. Regarding the GSE84437 dataset, the authors provided the expression matrix after normalization. The expression matrix of GSE84437 was then log2 transformed. Raw transcriptome and clinical data of immunotherapy cohort (IMvigor210) were retrieved using R package “IMvigor210CoreBiologies”.

Single cell-seq data from GSE183904 were selected for further analysis [19]. Our quality control standard is that each gene is expressed in 3 or more cells, and each cell has 300–5000 genes expressed. Cells with mitochondrial RNA percentages of > 20 were filtered out. We use the DoubletFinder package to remove the doublets cell [20]. Before clustering analysis, we performed canonical correlation analysis (CCA) to remove batch effects between different samples. We selected the top 3000 variably expressed genes as features for subsequent dimensionality reduction and cluster analysis using the FindVariableGenes function in Seurat. In using principal component analysis, we selected 30 principal components for subsequent analysis. The best resolution of 0.8 was chosen by us for cell clustering. Marker genes for various cell subtypes are obtained through the FindAllMarkers function with the parameter: logfc.threshold = 0.25, min.pct = 0.25 in Seurat. Since the cell types of stromal cells and myeloid cells could not be clearly distinguished in the first clustering, we extracted these cells for secondary clustering and performed cell type annotation.

### Scoring assessment of gene sets at bulk and single-cell resolution

In bulk data, we estimate gene set scores using the GSVA method in the GSVA package [21]. In single-cell data, we use gene set scoring methods named “singscore” collected in the irGSEA package for gene set scoring.

### Clustering and subcluster prediction

Based on the scores of 14 tumor features for each bulk sample calculated in the previous step, we clustered GC samples using consensus clustering with 1000 iterations and resampling of 80% using ConsensusClusterPlus package [22].

The single-cell sequencing used was not accompanied by bulk sequencing. We can think of bulk sequencing as measuring the total expression of each gene in all cells in a tumor tissue. Therefore, after the quality control of the single-cell expression matrix was completed, the average expression matrix of all cells in each sample was calculated to estimate the bulk-level expression of a single sample. Based on the prediction of the three clusters of TCGA-STAD dataset, we divided the TCGA-STAD dataset into two parts according to 7:3 using the createDataPartition function from the caret package. The proportions of the three clustering samples in two parts were consistent with original cohort. The former part is used as the training set, and the latter part is used as the validation data set. The single-cell samples were then subjected to subtype prediction based on the model trained on the training set. The above process uses the XGBoost algorithm [23]. We also performed subtype predictions for other tumors in TCGA, in the same way as described above.

### Evaluation of infiltrating immune cells in the TME

The CIBERSORT algorithm (https://cibersortx.stanford.edu/) was used to estimate the proportion of 22 immune cell types in GC samples with 1000 permutations of batch-corrected mode, relative mode and b-mode [24]. We use the ESTIMATE algorithm to estimate stromal score and immune score in GC [25].

### Establishment of a prognostic risk model for gastric cancer associated with tumor characteristics

In order to explore whether these 14 cancer functional states can predict the prognosis of gastric cancer, we used lasso-cox regression analysis to screen the most optimal cancer functional states, and then established a prognostic risk model in TCGA-STAD cohort using the glmnet package of R [26]. Based on the important features and weight coefficients obtained by the previous model, we calculated the risk score of each sample in the GPL570 cohort and GSE84437. The optimal cutoff value was confirmed by maxstat package. Overall survival curves were estimated by the Kaplan–Meier method (KM), and differences in survival were assessed by log-rank test.

### Additional analysis of single-cell RNA-sequencing data

Because the R version of scenic analysis is very time-consuming, we first pergormed pyscenic on the python platform to process single-cell data, and then use the R version of scenic to analyze the calculated files [27]. We identified multiple cell types in single-cell analysis and used the MuSiC package to estimate cell fraction in bulk samples [28]. We used SCORPIUS to infer cellular trajectories [29].

### Additional bioinformatic and statistical analyses

Half of the maximal inhibitory concentration (IC50) was estimated by the R package prophet. Connectivity Map (CMap, https://clue.io/) was used to predict candidate small molecules based on differentially expressed genes. ICB responses were predicted using the TIDE algorithm (http://tide.dfci.harvard.edu). Statistical differences not specifically stated were set at P < 0.05.

## Result

### Cancer functional states identify three GC subtypes

**Fig. 1:**
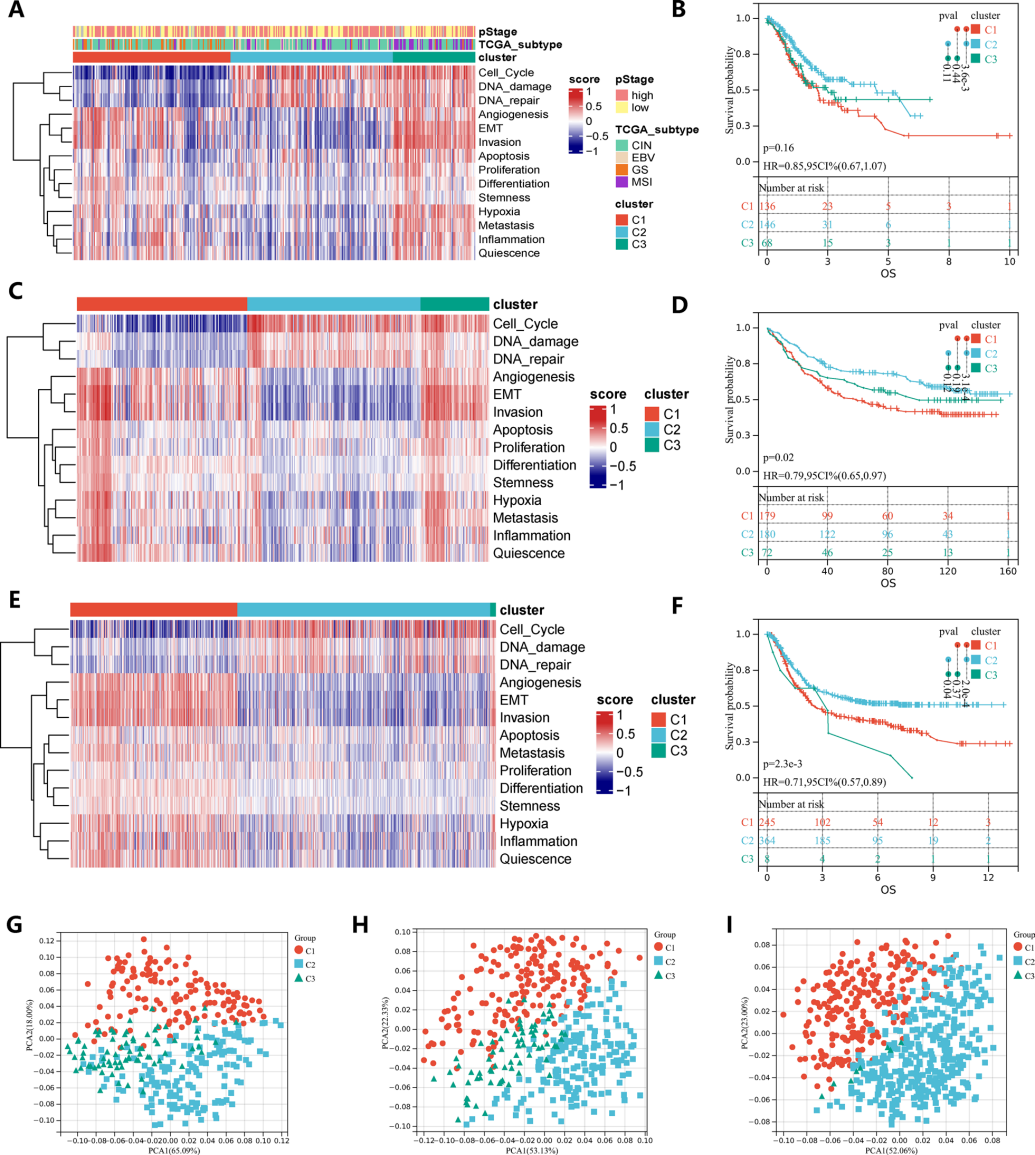
Three subtypes of gastric cancer based on cancer functional states. **A** Consensus clustering was performed to divide the TCGA-STAD cohort into three gastric cancer subtypes based on the cancer functional status scores calculated by the GSVA method. **B** Kaplan–Meier curves of OS in TCGA-STAD cohort between three subtypes. **C** Consensus clustering was performed to divide the GSE84437 cohort into three gastric cancer subtypes. **D** Kaplan–Meier curves of OS in GSE84437 cohort between three subtypes. **E** Consensus clustering was performed to divide the GPL570 meta cohort into three gastric cancer subtypes. **F** Kaplan–Meier curves of OS in GPL570 meta cohort between three subtypes. **G**, **H** and **I** PCA confirmed that the three subtypes of three cohorts could be distinguished significantly

Based on the 14 cancer functional status scores calculated by the GSVA method, we divided the TCGA-STAD dataset into 3 subtypes using the consensus clustering method (Fig. 1A, Additional file 2: Table S1). Principal component analysis found that the cluster 1 (C1) and the cluster 2 (C2) could be significantly separated, while the cluster 3 (C3) was distributed between the two subtypes, and it seemed that C3 was a category of transition features (Fig. 1G). We found that C3 is more like a cluster of the same characteristics picked from C1 and C2 (Additional file 1: Fig S1). KM analysis shows that C1 tended to have a worse survival prognosis, C2 has the best survival. The C3 has no significant survival difference, but the trend of the curve shows that the overall survival time of C3 is between C1 and C2 (Fig. 1B). C1 had low expression of cell cycle, DNA damage and DNA repair, and the remaining 11 cancer functional states had low levels. C2 is just the opposite of C1. C3 has high level in all cancer functional states (Fig. 1A). Similar results were also observed in the GPL570 dataset and GSE84437 (Fig. 1C–F, H, I).

### Signaling pathways and immune cells of three GC subtypes

**Fig. 2:**
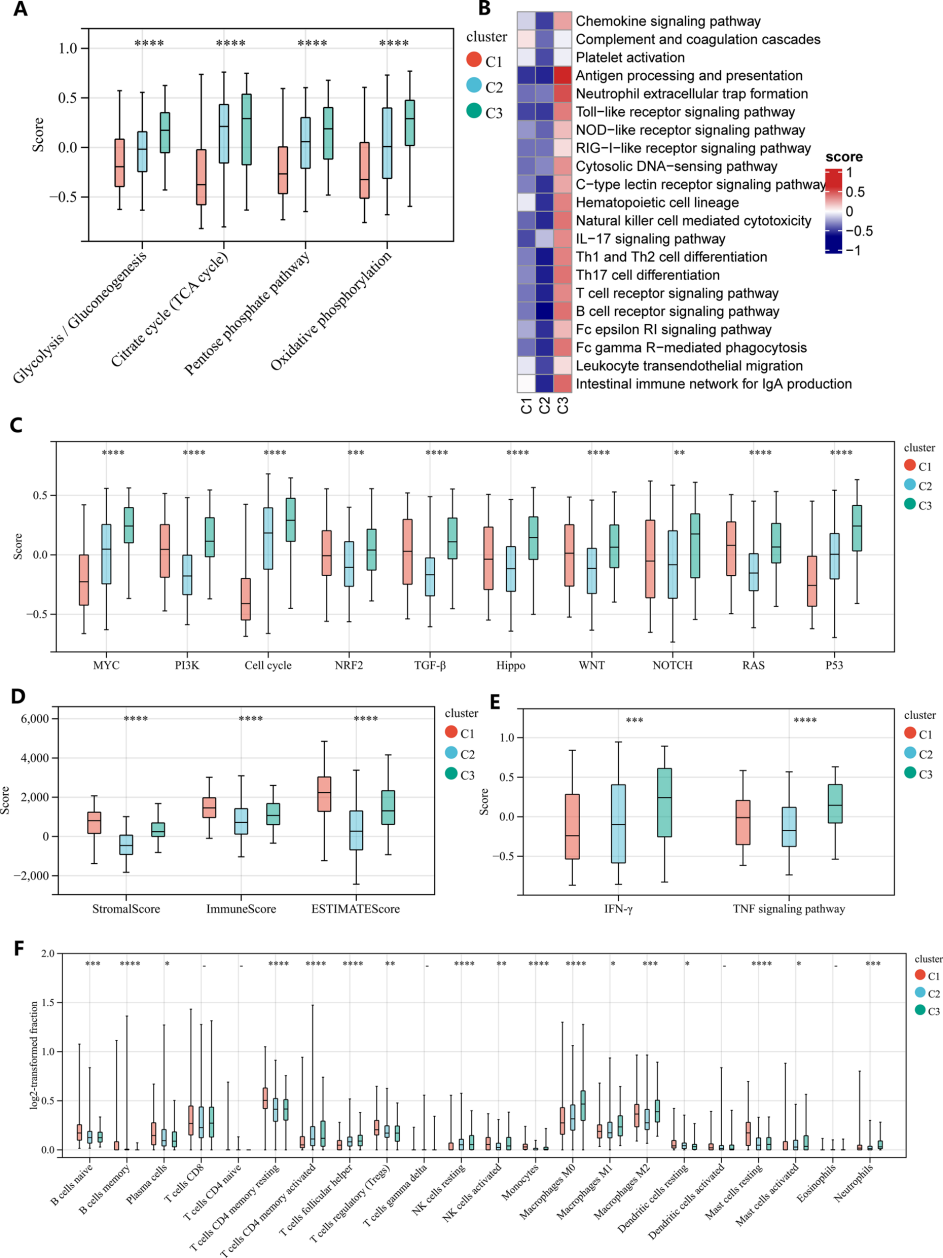
Immune and signaling pathway heterogeneity among the three subtypes. **A** Comparison of four energy metabolism pathways among three gastric cancer subtypes. **B** Comparison of KEGG immune pathways among three gastric cancer subtypes (The color of the squares indicates the high and low average scores of all samples within each subtype). **C** Comparison of 10 cancer-related pathways among three gastric cancer subtypes. **D** Comparison of Stromal score, Immune score and ESTIMATE score among three gastric cancer subtypes. **E** Comparison of IFN-γ and TNF signaling pathway among three gastric cancer subtypes. **F** Relative proportion of 22 infiltrating immune cells estimated by CIBERSORT among three gastric cancer subtypes. The above analyses are all performed in TCGA-STAD

To explore the functional status among the three subtypes, we calculated scores for all Kyoto Encyclopedia of Genes and Genomes (KEGG) signaling pathways using the GSVA method. Interestingly, almost all metabolic related KEGG signaling pathways were highly expressed in C3, suggesting that cells of C3 are in the status of high levels of metabolism (Additional file 1: Fig S2). The activities of the 4 energy metabolic pathways (glycolysis/gluconeogenesis, citrate cycle, pentose phosphate pathway and oxidative phosphorylation) are: C1 < C2 < C3 (Fig. 2A). Immune-related pathway analysis showed that C3 had the highest immune activity, followed by C2, and C1 was the lowest (Fig. 2B). In addition, we also analyzed the expression of 10 cancer-related signaling pathways, all of which are highly expressed in C3 (Fig. 2C).

The immune and stromal scores were calculated by the ESTIMATE algorithm. Similar to the results of the signaling pathway analysis, these data confirmed that C1 and C2 had the highest and lowest immune score, stromal score and ESTIMATE score, respectively (Fig. 2D). In addition, we also used the CIBERSORT algorithm to estimate the proportions of 22 immune cells in GC samples. C1 was characterized by high B cells naive, B cells memory, plasma cells, T cells CD4 memory resting, T cells regulatory (Tregs), dendritic cells resting and mast cells resting (Fig. 2F). C3 was remarkably rich in T cells CD4 memory activated, T cells follicular helper, NK cells resting, NK cells activated, macrophages M0, macrophages M1, macrophages M2 and neutrophils (Fig. 2F). C2 is characterized by low infiltration of all immune cells. Furthermore, to assess the immune cytolytic activity of GC samples, we analyzed the differences in IFN-γ and TNF signaling pathway among the three subgroups. Interestingly, C3 was the highest in both scores, indicating that C3 has the strongest immune cytolytic activity (Fig. 2E).

### Genomic alterations of three GC subtypes

**Fig. 3:**
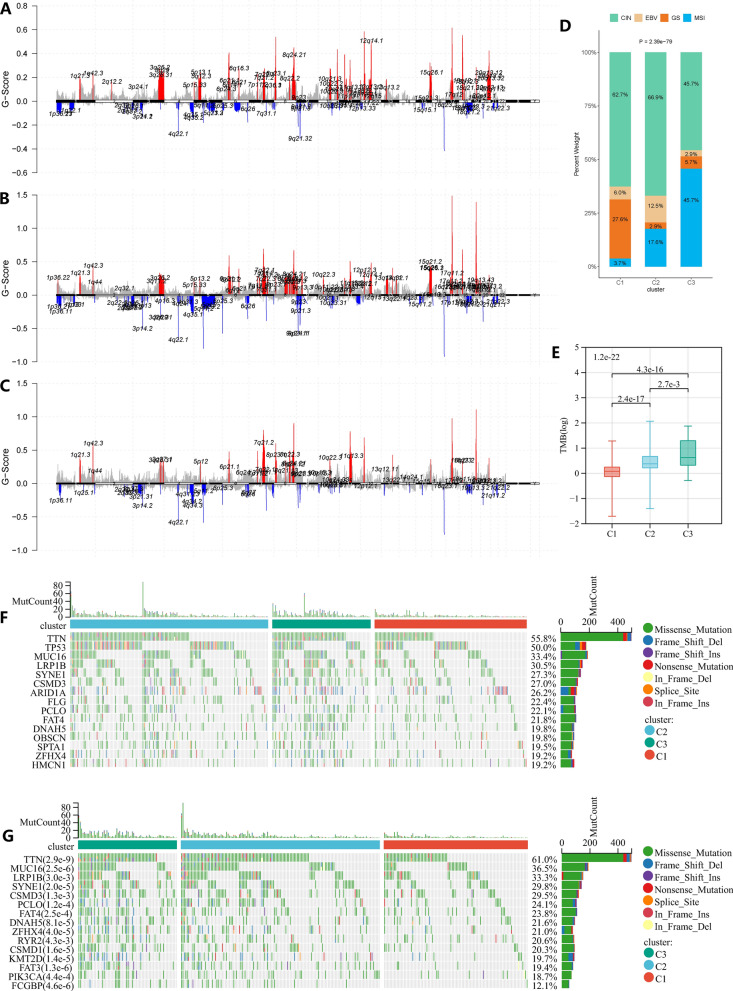
Comparison of genomic alterations in three subtypes in the TCGA-STAD cohort. **A**, **B** and **C** Comparison of the somatic copy number variations among three gastric cancer subtypes. **D** Comparison of TCGA gastric cancer subtypes among three gastric cancer subtypes. **E** comparison of tumor mutation burden among three gastric cancer subtypes. **F** Top 20 mutated genes in all gastric cancer patients. **G** Top 20 differentially mutated genes between gastric cancer subtypes

To explore the genomic differences between three subtypes, we analyzed the CNV profile of TCGA-STAD with a threshold of q < 0.05. After removing germline CNVs, we found that C3 had lower levels of arm- and focal-level CNVs compared to C1 and C2 (Fig. 3A–C). Furthermore, C1 and C2 had a higher proportion of chromosomal instability (CIN) patients relative to C1 (Fig. 3D). The above results demonstrate that C3 has better genomic stability. This was also demonstrated by differences in the number of genes affected by CNV among the three subtypes.

Next, we analyzed the differences in gene mutations among the three subtypes. We analyzed the top 20 genes with different mutation frequency among the three subtypes, 13 of which (TTN, MUC16, FAT3, LRP1B, SYNE1, CSMD3, PCLO, FAT4, DNAH5, ZFHX4, RYR2, CSMD1 and KMT2D) were also in the top 20 mutated genes for all GC samples (Fig. 3F, G). We found that C3 has a higher mutation frequency than C1 and C2, and C2 has a higher mutation frequency than C1. This is consistent with the distribution of microsatellite instable (MSI) patients among the three subtypes (Fig. 3D). This was also supported by differences in tumor mutational burden (TMB) among the three groups (Fig. 3E). These results indicated that gene mutations may be associated with the phenotype of GC subtypes.

### Cancer functional states score as a marker for prognosis

**Fig. 4:**
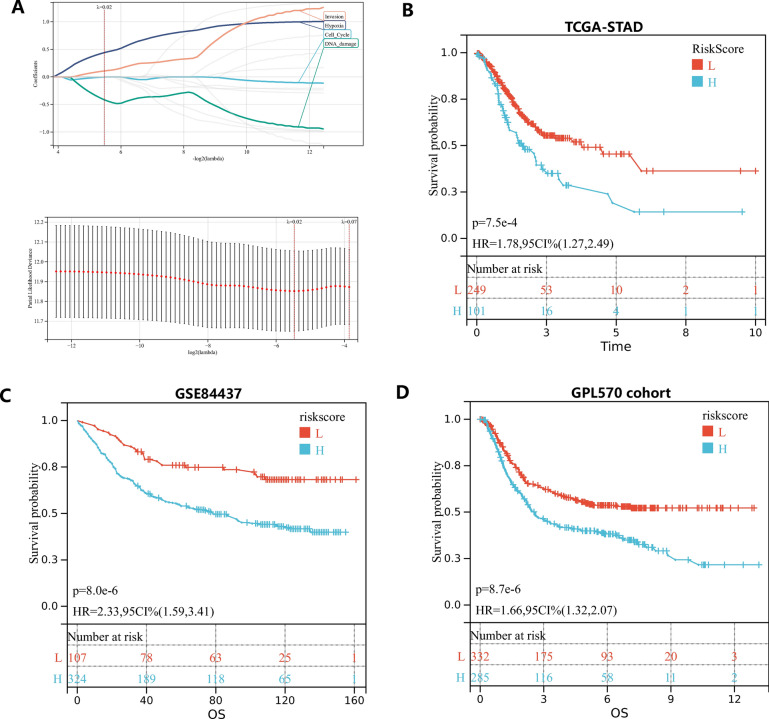
Construction of caner functional states associated prognostic model. **A** Partial likelihood deviance for the lasso regression and Lasso regression analysis. **B** Patients were divided into high-risk and low-risk subgroup based optimal cutoff, Kaplan–Meier analysis demonstrated that patients with higher risk score exhibited worse overall survival in TCGA-STAD. **C**, **D** The prognostic difference was validated in GSE84437 and GPL570 cohort

Significant OS differences between subtypes were observed, so we thought whether the cancer functional status score could be a prognostic marker. The Lasso-Cox algorithm was used to identify the most robust cancer functional states pathways for prognosis prediction after obtaining 14 gene set scores of all samples from the TCGA-STAD cohort (Fig. 4A). Finally, risk models associated with cancer functional status pathways were developed as fellow: -0.00135455854945771*Cell Cycle-0.416041346360079*DNA damage + 0.440674355732646*Hypoxia + 0.10833269488575*Invasion. As shown in Fig. 4B, KM analysis demonstrated that patients with higher risk score exhibited worse overall survival in TCGA-STAD (HR = 1.78, 95% CI = 1.27–2.49, P = 7.5e-4). In order to validate the stability of the model, we performed analysis in two additional cohorts. Similar results were also observed in GPL570 cohort and GSE84437(GPL570 dataset: HR = 1.66, 95% CI = 1.32–2.07, p = 8.7e-6; GSE84437: HR = 2.33, 95% CI = 1.59–3.41, p = 8.0e-6; Fig. 4C, D).

### Stromal cells dominate the cancer functional state

**Fig. 5:**
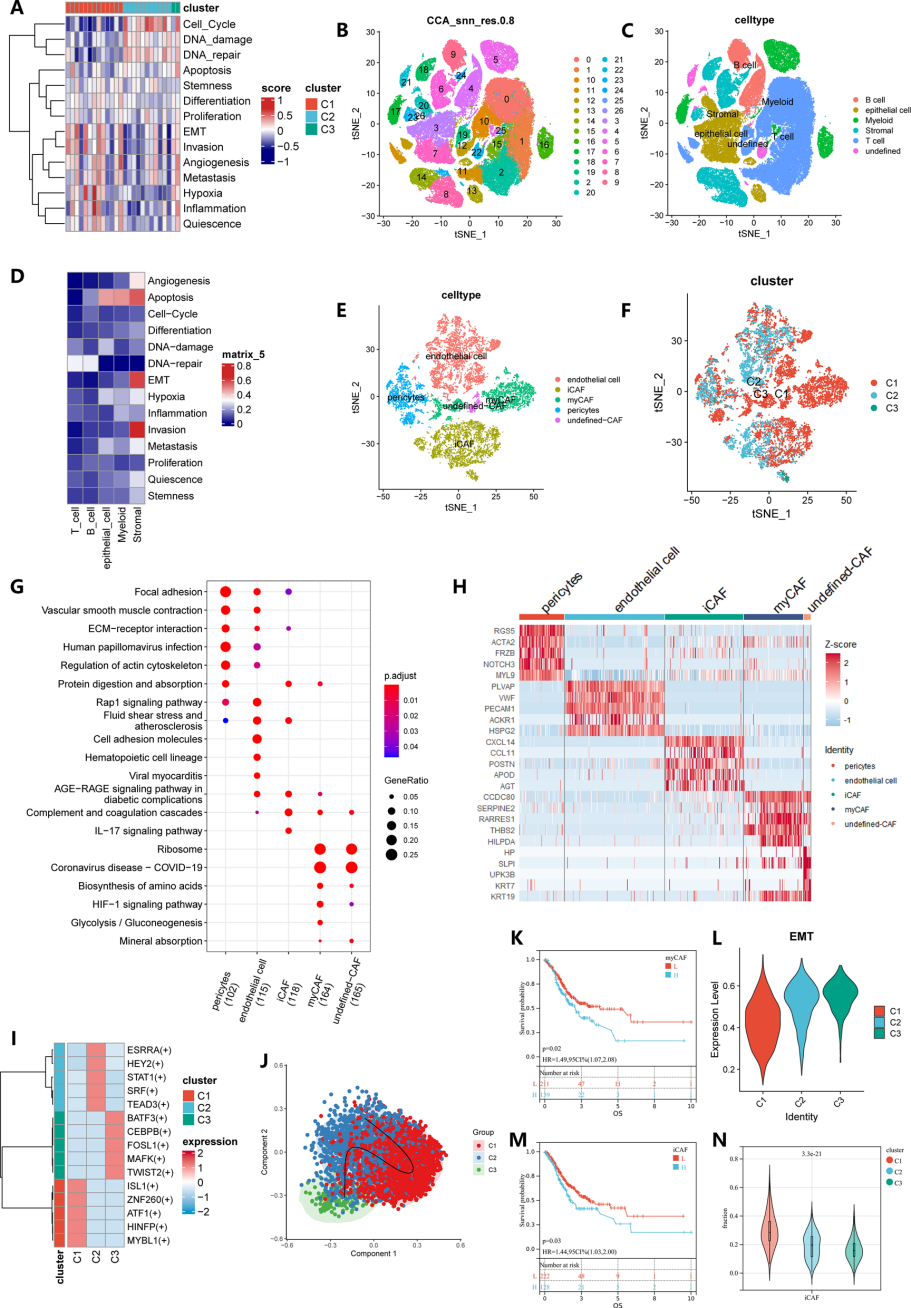
Identification of all cell subtypes in single-cell samples and comparison of stromal cells between three gastric cancer subtypes in GSE183904. **A** Heatmap displays of 14 cancer functional states for three subtypes of predicted single-cell samples. **B**, **C** The tSNE plot of single cells profiled in the presenting work colored by 27 cell clusters and major cell types. **D** 14 cancer functional states score between three gastric cancer subtypes. **E**, **F** The tSNE plot of stromal cells profiled colored by major stromal cell types and three subtypes. **G** KEGG pathway enriched in marker genes of five stromal cells. **H** Top 5 marker genes of 5 stromal cells. **I** Heatmap shows normalized activity of top 5 TF regulons in three subtypes predicted by pySCENIC. **J** Potential trajectory of iCAF inferred by SCORPIUS. **K** Kaplan–Meier analysis of high- and low myCAF infiltration in TCGA-STAD. **L** Comparison of EMT activity of myCAF between three gastric cancer subtypes. **M** Kaplan–Meier analysis of high- and low iCAF infiltration in TCGA-STAD. **N** Comparison of iCAF infiltration between three gastric cancer subtypes

Since tumor tissue is not composed of single type of cells, including epithelial cells, immune cells, and mesenchymal cells, it is necessary to explore the differences between various cell types among GC subtypes. The original data contained many tumor-adjacent normal samples and some un-primary tumor samples. But we selected the primary tumor samples for analysis. Detailed clinical and pathological information is provided in Additional file 2: Table S2. After following our standard quality control, we obtained 93583 cells of 26 GC samples for subsequent analysis. We thought that by calculating the average gene expression of all cells in each single-cell sample, we can obtain approximate bulk-level sequencing results. We first randomly divide TCGA-STAD into two parts according to 7:3, the former is used as the training cohort, and the latter is used as the validation cohort. The model was then trained in the training dataset based on the 14 cancer functional status scores, which had an area under the curve (AUC) value of 1 in the training dataset. Shockingly, in the validation cohort, the AUC also reached 0.9909. Then we used the trained model to predict the GC subtypes of the single-cell cohort samples, and finally got 13 patients of C1, 11 of C2, and 2 of C3 (Additional file 2: Table S3). The distributional characteristics of cancer functional status were also consistent with those of the TCGA-STAD cohort (Fig. 5A), which proves the robustness of the classifier. Based on res of 0.8, a total of 27 cell clusters are obtained (Fig. 5B). We then identified five major cell types, including epithelial cells, T cells, B cells, stromal cells, and myeloid cells (detailed markers in Additional file 2: Table S4, Fig. 5C). The heatmaps of these cancer functional status scores showed predominantly high scores in stromal cells (Fig. 5D). So, we next analyzed the differences of stromal cells among the three subtypes.

The stromal cells can be re-clustered into 14 clusters with res of 0.3. We annotated these stromal cells as pericytes, myo-cancer associated fibroblasts (myCAFs), undefined cancer-associated fibroblasts (undefined-CAFs), inflammatory cancer-associated fibroblasts (iCAFs), and endothelial cells (detailed markers in Additional file 2: Table S5; Fig. 5E). Remarkably, myCAF is a specific cell type of the C1 subtype, implying that myCAF may be involved in the formation of C1 subtype-related characteristic (Fig. 5E, F). We performed KEGG pathway enrichment analysis on the marker genes of myCAF. The results suggested that myCAF was in a relatively active state, which may be related to its tumor-promoting effect and is consistent with C1 having the worst prognosis (Fig. 5G, H). KM analysis also suggested that GC patients with high myCAF infiltration have poor OS (p = 0.02; Fig. 5K). We investigated developmental trajectories in endothelial cells, iCAF and pericytes, respectively. But in endothelial cells and pericytes, we did not find obvious trajectory variety among the three clusters. Only the developmental pattern of C1-C2-C3 presented in iCAF (Fig. 5J). We also performed a scenic analysis in iCAF. The results indicate that MAFK and TWIST2 are highly activated in C3 (Fig. 5I). Previous studies have shown that these two transcription factors induce EMT and then promote tumor progression [30, 31], which is consistent with the high EMT score in C3 (Fig. 5L). At the same time, we found that high iCAF infiltration means worse OS, which may be related to the ability of iCAF to promote tumor progression (Fig. 5M). We also found that iCAF infiltration was also highest in C1 and lowest in C3 among the three subtypes in the TCGA-STAD cohort (Fig. 5N).

### C3 has better response to immunotherapy

**Fig. 6:**
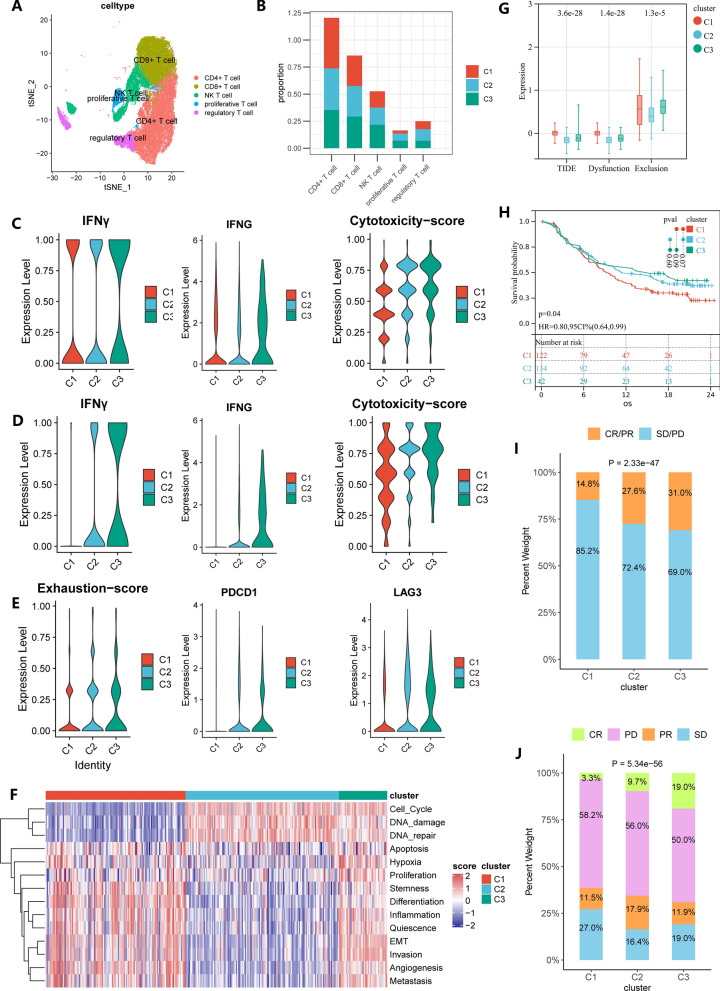
Comparison of T cells and immunotherapy response between three gastric cancer subtypes in GSE183904. **A** The tSNE plot of T cells profiled colored by 5 T cell types. **B** Comparison of 5 T cells of three subtypes. Comparison of IFN-γ signaling pathway, IFNG gene, and cytotoxicity score between three subtypes in CD8 + T cell (**C**) and NK T cell (**D**). **E** Comparison of exhaustion score and PDCD1, and LAG3 in NK T cell between three subtypes. **F** Heatmap displays of 14 cancer functional states for three subtypes in the IMvigor210 cohort. **G** Comparison of TIDE score, dysfunction score and exclusion score between three subtypes in TCGA-STAD. **H** Kaplan–Meier curves of OS in the IMvigor210 cohort between three subtypes. **I**, **J** Proportion of patients with response to ICI immunotherapy in three subtypes in the IMvigor210 cohort. CR, complete response; PR, partial response; SD, stable disease; PD, progressive disease. CR/PR was identified as responder, and SD/PD was identified as non-responder

We annotated T cells as CD4 + T cells, CD8 + T cells, NK T cells, regulatory T cells (Tregs) and proliferative T cells (detailed markers in Additional file 2: Table S6, Fig. 6A). Since T cells play an important role in antitumor immunity, we next investigated the characteristics heterogeneity of T cell between three subtypes. There was no significant difference in the number of various types of T cells among the three subtypes (Fig. 6B). To investigate the differences in the cytotoxicity of the three subtypes of T cells, we focused on CD8 + T cells and NK T cells. We found that C3 had stronger tumor-killing activity, as evidenced by either the IFN-γ pathway and the expression of the IFNG gene or the cytotoxicity score (Fig. 6C, D). And NK T cells of C3 seem to have more significant tumor-killing activity than C1 and C2 (Fig. 6C, D).

Now that C3 has the strongest antitumor ability, we next analyzed whether the three GC subtypes had different responses to immunotherapy. Among CD8 + T cells, C3 had higher exhaustion scores and immune checkpoint gene expression (PDCD1, LAG3), which proved that C3 may have a better response to immunotherapy (Fig. 6E). We found that at the bulk level, C3 also had higher TIDE scores than C1 and C2, which is consistent with our findings at single-cell resolution (Fig. 6G). We also analyzed differences between the three subtypes in a cohort of urothelial carcinomas undergoing immunotherapy. First, we typed the patients in the IMvigor210 cohort as three subtypes based on the previously established subtype prediction model, and the three subtypes also had the same characteristic distribution as the previous gastric cancer cohort (Fig. 6F). The three subtypes also had the same survival differences as the GC cohort (Fig. 6H). The proportion of immunotherapy responders was significantly higher in C3 compared with C1 and C2, especially the proportion of complete responders (Fig. 6I, J).

### Subtype-related treatment strategies

**Fig. 7:**
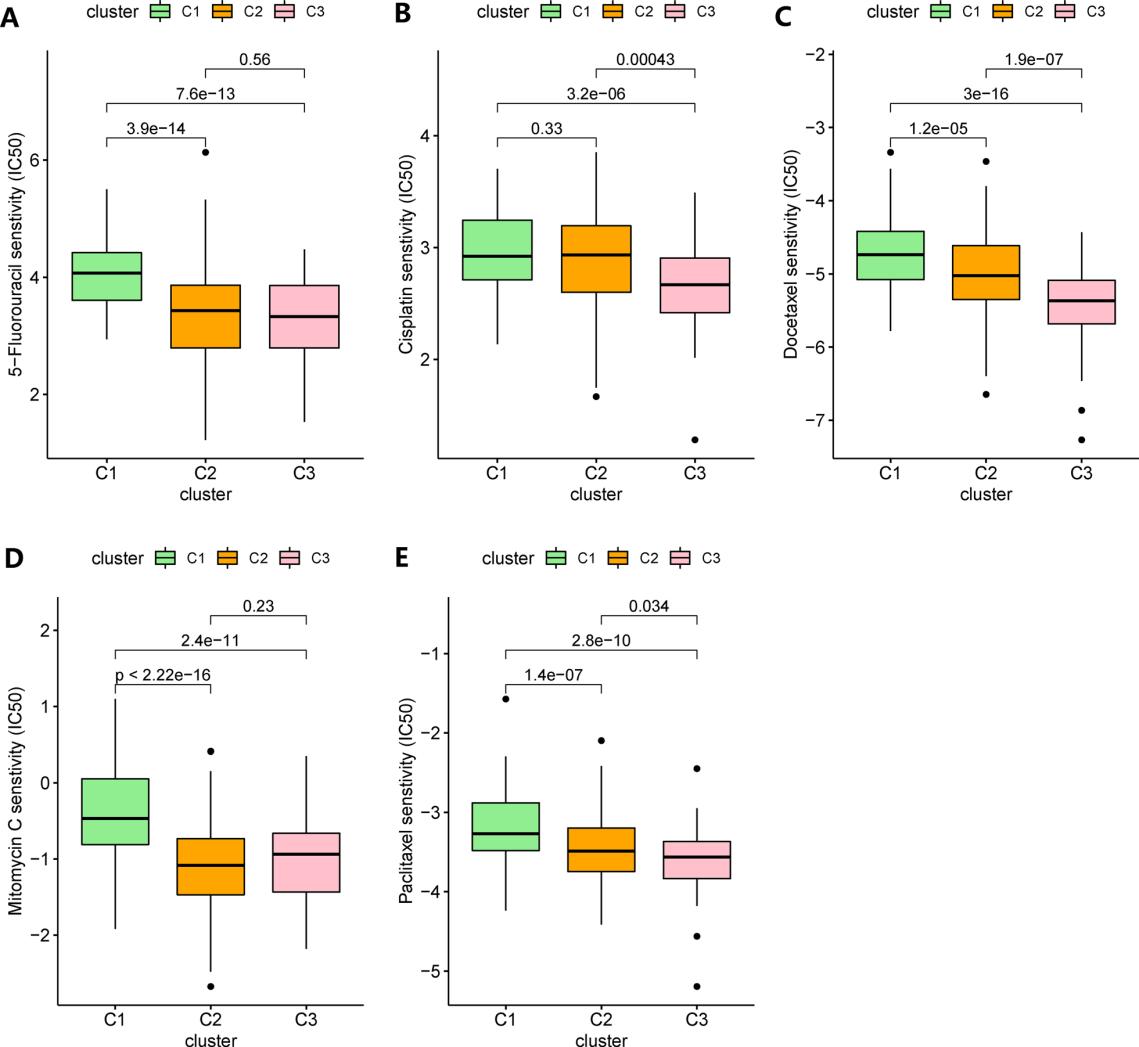
The estimation of chemotherapy response for three gastric cancer subtypes in TCGA-STAD. **A–E** The chemotherapy response of three subtypes for five common chemotherapy drugs

Chemotherapy is an important strategy in cancer treatment. The Cancer Genome Project (CGP) database was used to predict chemotherapeutic response. The results showed that 5-Fluorouracil, cisplatin, docetaxel, mitomycin C and paclitaxel were more suitable for patients of C1 (Fig. 7). We used the CMap database to predict drugs with specific therapeutic effects on the three subtypes. We predicted many drugs specific to the three subtypes (Additional file 2: Table S7).

## Discussion

In this study, we proposed a classification method of gastric cancer based on 14 cancer functional status, and based on this gastric cancer was divided into three subtypes. This classification method has also been shown to be stable in multiple datasets. We elucidate the characteristics of the three subtypes in detail. C1 is characterized by high levels of tumor progression-related cancer functional status, worst survival outcomes, low metabolic level, high infiltration of immunosuppressive cells, high CNV, and low TMB. C2 is characterized by low levels of tumor progression-related cancer functional status, favorable prognosis, moderate metabolic level, low immune cell infiltration, high CNV, and moderate TMB. Then, C3 is characterized by the high level of all cancer functional status, high metabolic level, low CNV, high TMB, high infiltration of immune cells with high cytotoxicity, and better response to immunotherapy. Many differences between subtypes were also validated by single-cell data. These differential characteristics among the three subtypes suggest that our GC classification should be taken into consideration when developing personalized therapy.

We divided GC into three subtypes based on levels of 14 types of cancer functional status. This classification system was not only consistent with TCGA-STAD performance in the GPL570 cohort and GSE84437, but also with the characteristics of the three subtypes of the IMvigor210 cohort. To verify whether our classification model can be applied to other tumors, we performed subtype predictions for all tumors in the TCGA database. The results showed that the subtypes of cervical squamous cell carcinoma and endocervical adenocarcinoma, colon adenocarcinoma, lung squamous cell carcinoma, and thymoma had the same prognostic differences as the subtypes of GC (Additional file 1: Fig S3). We also found significant prognostic differences among the three subtypes for 12 tumors, but not consistent with differences among gastric cancer subtypes (Additional file 1: Fig S4).

It is worth noting that only cell cycle, DNA damage and DNA repair are highly expressed in C2, while many other pathways, such as angiogenesis, EMT, hypoxia, invasion, metastasis, and stemness related to malignant characteristics of tumors, are low-expressed. This suggests that C2 is a relatively low-malignancy GC subtype, which is consistent with the best prognosis of C2. Although C2 is characterized by low immune cell infiltration, single-cell analysis suggests that C2 possesses T cell tumor-killing capacity second only to C3. Although both C1 and C3 have high expression of tumor malignant characteristics, C1 has high infiltration of immunosuppressive cells, which makes the prognosis of C1 poor. This was also demonstrated by the lowest tumor-killing capacity of T cells of C1 at single-cell resolution. C3 is characterized by high expression in all cancer functional states, and C3 is at high levels in almost all metabolic pathways. The immune cell infiltration of C3 is also more abundant, and the tumor killing ability is also the strongest. This probably means that C3 is active. Immune checkpoint molecules mainly exist on the surface of various immune cells, and the ligands on the surface of other immunosuppressive cells can inhibit the cytotoxic effect of T cells after binding to them [32, 33. Interestingly, our single-cell analysis found that T cells in C3 had higher expression of immune checkpoint genes and immune exhaustion scores, so we thought that C3 could benefit from immunotherapy. We also validated our conclusions in an immunotherapy cohort. It is worth noting that C3 not only had a better response to immunotherapy, but also had a higher sensitivity to the five chemotherapeutic agents.

A number of previous studies had also identified various subtypes of gastric cancer [4, 5] [34–8]. Compared to their classification methods, ours has some strengths. First, we classify based on 14 functional states of cancer, which makes our classification factors more comprehensive. The second is that we have a more comprehensive analysis of the differences between the subtypes, especially our elucidation of these differences at single-cell resolution.

## Conclusion

In conclusion, we divided gastric cancer into three subtypes based on 14 cancer functional states. We elucidated the differences of characteristics among the three subtypes from the level of signaling pathways, genomic alterations, and single-cell levels. We found that C3 is more sensitive to a variety of chemotherapeutic agents and can benefit from immunotherapy. This provides new insights into the clinical treatment of gastric cancer.

## Supplementary Information

**Additional file 1**: **Figure S1.** The sankey diagram of sample variation with 2, 3 and 4 cluster numbers. **Figure S2**. Comparison of KEGG pathways among three gastric cancer subtypes. **Figure S3**. Kaplan-Meier curves of OS in CESC, COAD, LUSC and THYM cohort between three subtypes. **Figure S4**. Kaplan-Meier curves of OS in ACC, BRCA, HNSC, KICH, KIRP, LGG, LIHC, LUAD, MESO, SARC and UCEC cohort between three subtypes.

**Additional file 2**: **TableS1**. Subtype classification in TCGA-STAD. **TableS2**. Clinical information of the eight single-cell sequencing patients included in the study. **TableS3**. Predicted single-cell sample subtypes. **Table S4**. Marker genes for 5 types of cell classification annotation. **Table S5**. Marker genes for stromal cell classification annotation. **Table S6**. Marker genes for T cell classification annotation. **Table S7.1**. Drugs predicted in subtype C1 by the CMap database. **Table S7.2**. Drugs predicted in subtype C2 by the CMap database. **Table S7.3**. Drugs predicted in subtype C3 by the CMap database.

## Data Availability

The data that support the findings of this study are available in GEO (https://www.ncbi.nlm.nih.gov/geo/, GSE62254, GSE15459, GSE57303, GSE34942, GSE84437 and GSE183904), TCGA (https://portal.gdc.cancer.gov/repository, TCGA-STAD and other tumor cohorts), and the Supporting Information. Then, raw transcriptome and clinical data of immunotherapy cohort (IMvigor210) were retrieved using R package “IMvigor210CoreBiologies”.

## Abbreviations

GC: Gastric cancer
AJCC: American Joint Committee on Cancer
TCGA: The cancer genome atlas
ACRG: The Asian cancer research group
ICIs: Immune checkpoint inhibitors
SNP: Single nucleotide polymorphism
CNV: Copy number variations
TPM: Transcripts per million
GEO: Gene-expression omnibus
CCA: Canonical correlation analysis
KM: The Kaplan–Meier method
IC50: Half of the maximal inhibitory concentration
C1: Cluster 1
C2: Cluster 2
C3: Cluster 3
KEGG: Kyoto encyclopedia of genes and genomes
TMB: Tumor mutational burden
AUC: The area under the curve
myCAFs: Myo-cancer associated fibroblasts
undefined-CAFs: Undefined cancer-associated fibroblasts
iCAFs: Inflammatory cancer-associated fibroblasts
