# Supplementary material for: Cancer functional states-based molecular subtypes of gastric cancer
Source: J Transl Med. 2023 Feb 4;21:80. doi: 10.1186/s12967-023-03921-1 (PMC9899380; doi:10.1186/s12967-023-03921-1)
Supplement: Supplementary file 1 — Additional file 1: Figure S1. The sankey diagram of sample variation with 2, 3 and 4 cluster numbers. Figure S2. Comparison of KEGG pathways among three gastric cancer subtypes. Figure S3. Kaplan-Meier curves of OS in CESC, COAD, LUSC and THYM cohort between three subtypes. Figure S4. Kaplan-Meier curves of OS in ACC, BRCA, HNSC, KICH, KIRP, LGG, LIHC, LUAD, MESO, SARC and UCEC cohort between three subtypes. [file 12967_2023_3921_MOESM1_ESM.pdf]

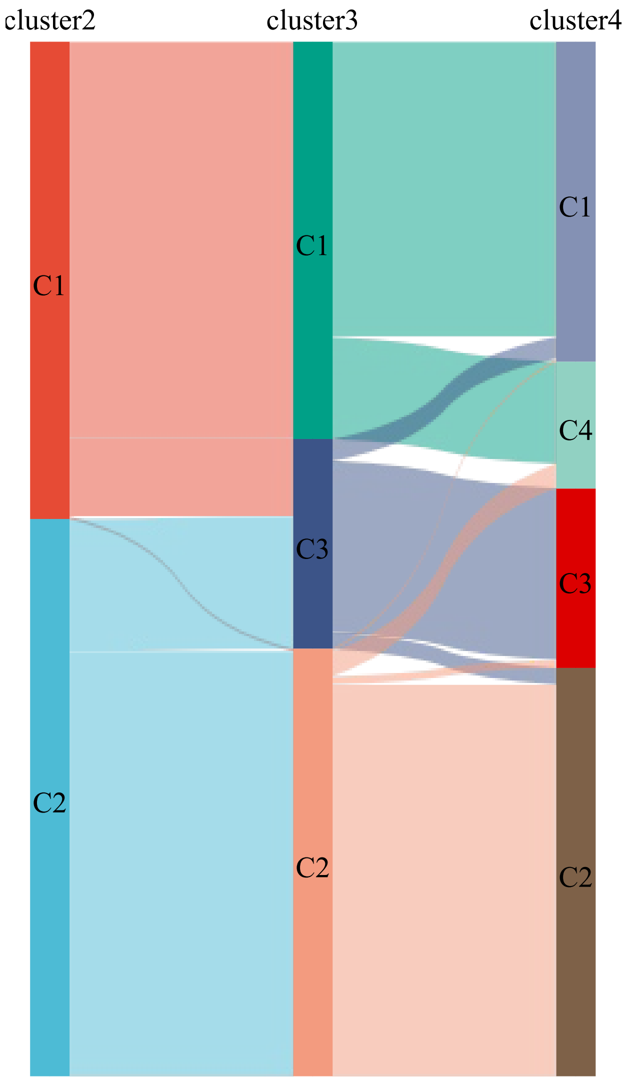

Figure S1. The sankey diagram of sample variation with 2, 3 and 4 cluster numbers.

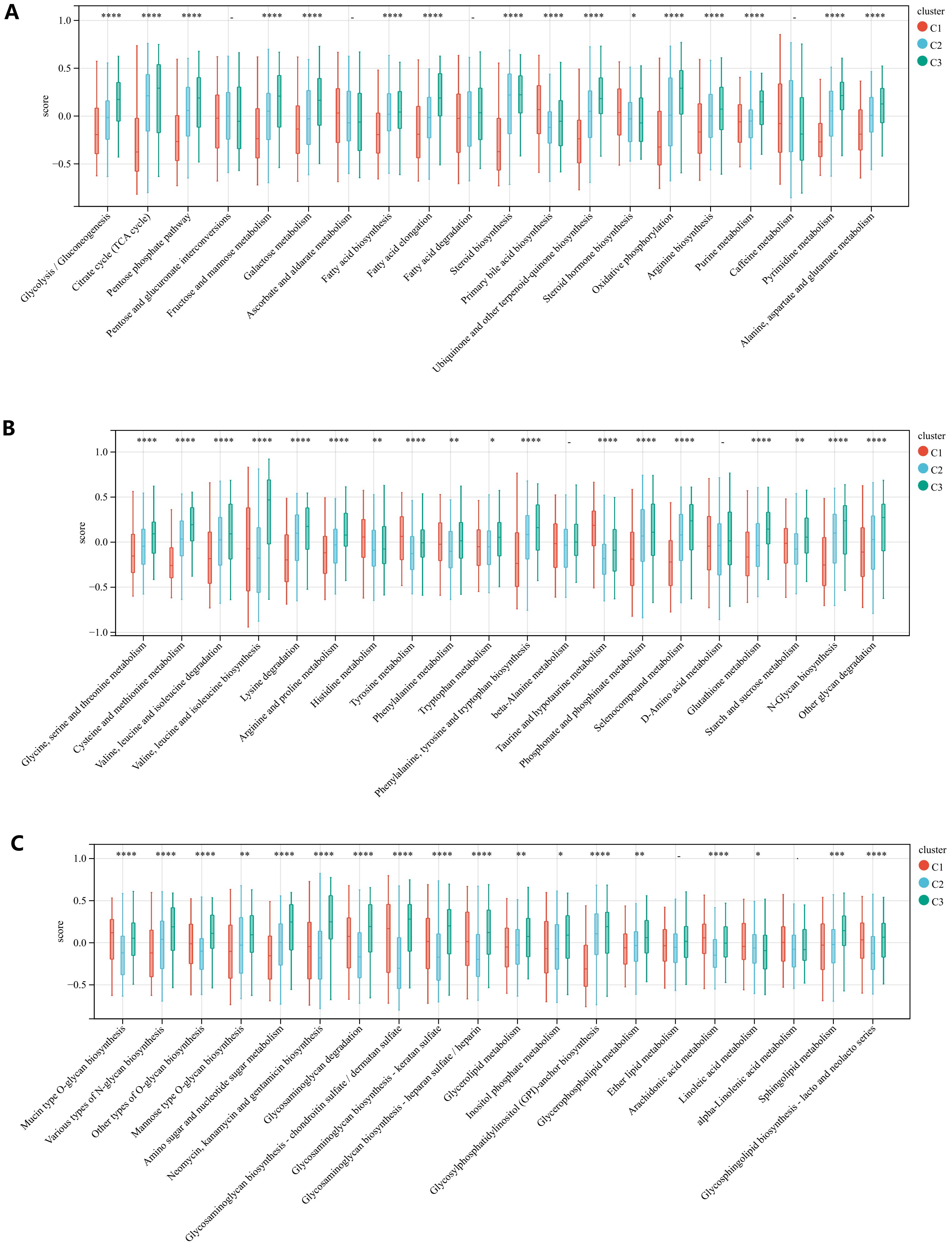
A
B
C
score
cluster
C1
C2
C3
1.0
0.5
0.0
−0.5
−1.0
Glycolysis / Gluconeogenesis
Citrate cycle (TCA cycle)
Pentose phosphate pathway
Pentose and glucuronate interconversions
Fructose and mannose metabolism
Galactose metabolism
Ascorbate and aldarate metabolism
Fatty acid biosynthesis
Fatty acid elongation
Fatty acid degradation
Steroid biosynthesis
Primary bile acid biosynthesis
Ubiquinone and other terpenoid-quinone biosynthesis
Steroid hormone biosynthesis
Oxidative phosphorylation
Arginine biosynthesis
Purine metabolism
Caffeine metabolism
Pyrimidine metabolism
Alanine, aspartate and glutamate metabolism
Glycine, serine and threonine metabolism
Cysteine and methionine metabolism
Valine, leucine and isoleucine degradation
Valine, leucine and isoleucine biosynthesis
Lysine degradation
Arginine and proline metabolism
Histidine metabolism
Tyrosine metabolism
Phenylalanine metabolism
Tryptophan metabolism
Phenylalanine, tyrosine and tryptophan biosynthesis
beta-Alanine metabolism
Taurine and hypotaurine metabolism
Phosphonate and phosphinate metabolism
Selenocompound metabolism
D-Amino acid metabolism
Glutathione metabolism
Starch and sucrose metabolism
N-Glycan biosynthesis
Other glycan degradation
Mucin type O-glycan biosynthesis
Various types of N-glycan biosynthesis
Other types of O-glycan biosynthesis
Mannose type O-glycan biosynthesis
Amino sugar and nucleotide sugar metabolism
Neomycin, kanamycin and gentamicin biosynthesis
Glycosaminoglycan degradation
Glycosaminoglycan biosynthesis - chondroitin sulfate / dermatan sulfate
Glycosaminoglycan biosynthesis - keratan sulfate
Glycosaminoglycan biosynthesis - heparan sulfate / heparin
Glycerolipid metabolism
Inositol phosphate metabolism
Glycosylphosphatidylinositol (GPI)-anchor biosynthesis
Glycerophospholipid metabolism
Ether lipid metabolism
Arachidonic acid metabolism
Linoleic acid metabolism
alpha-Linolenic acid metabolism
Sphingolipid metabolism
Glycosphingolipid biosynthesis - lacto and neolacto series

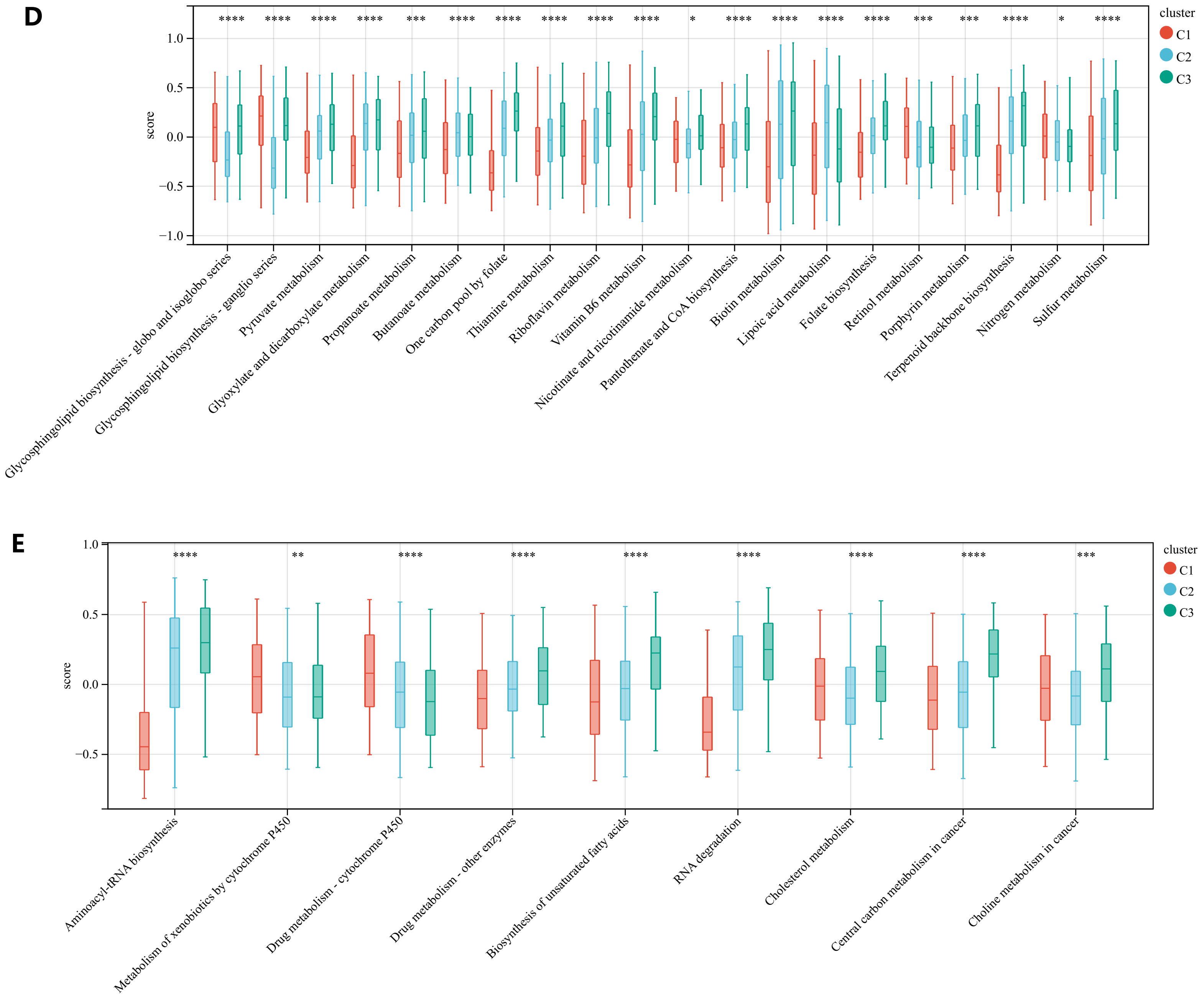

Figure S2. Comparison of KEGG pathways among three gastric cancer subtypes.

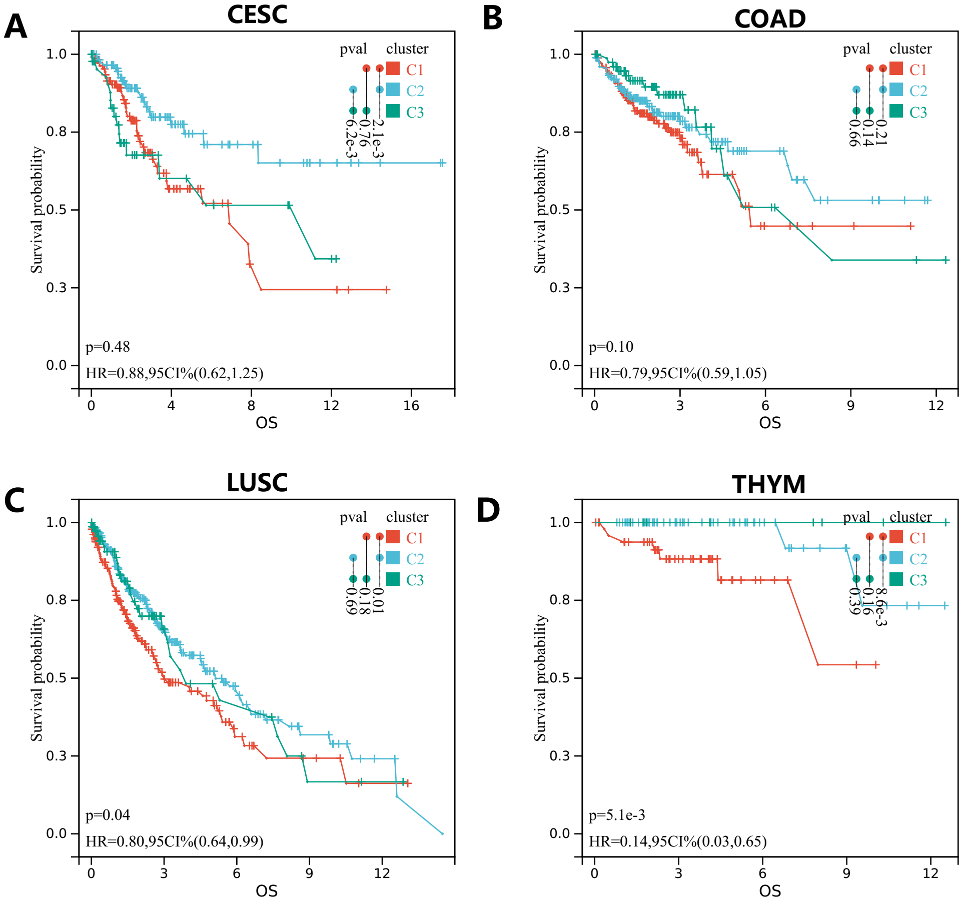

Figure S3. Kaplan-Meier curves of OS in CESC, COAD, LUSC and THYM cohort between three subtypes.

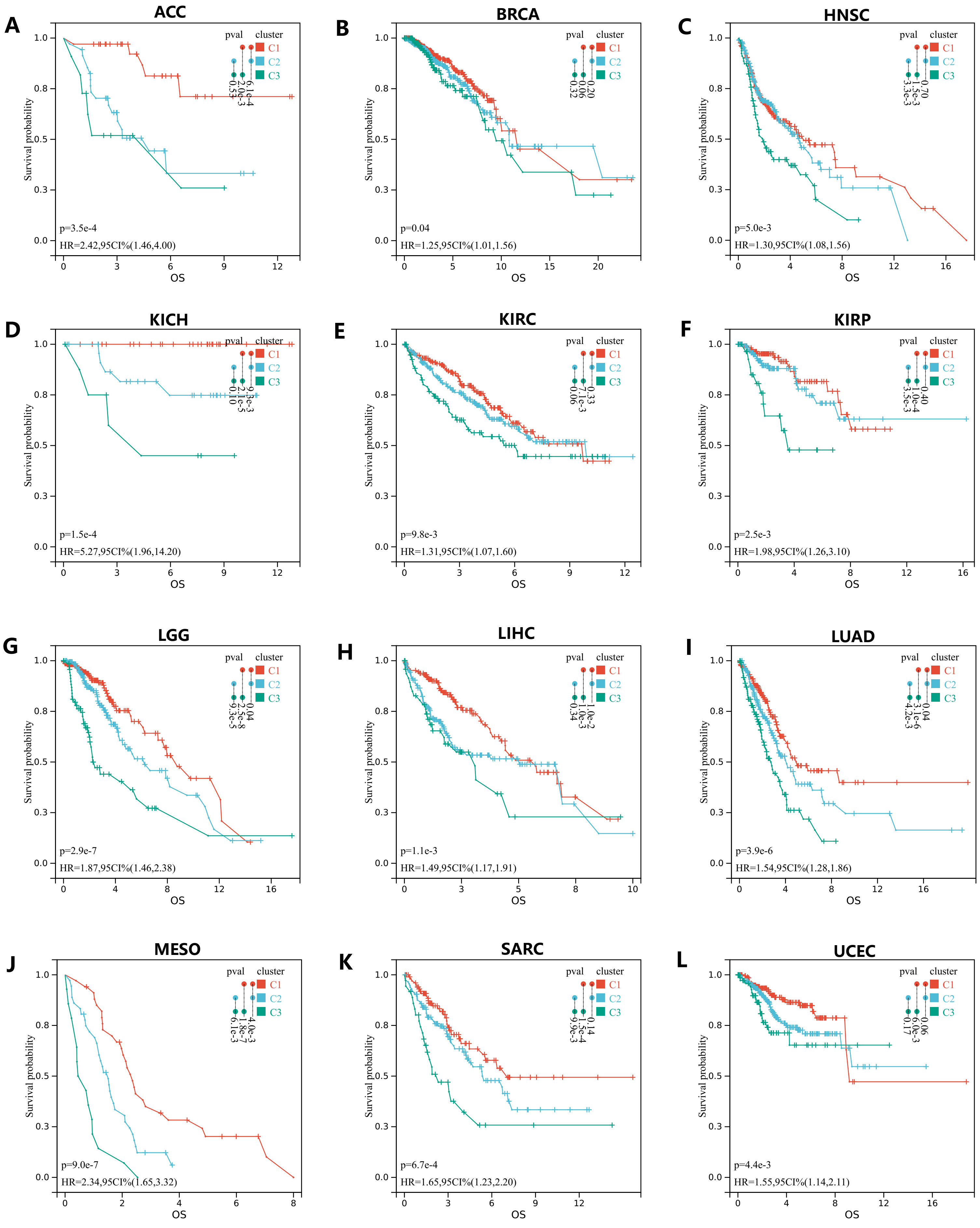

Figure S4. Kaplan-Meier curves of OS in ACC, BRCA, HNSC, KICH, KIRC, KIRP, LGG, LIHC, LUAD, MESO, SARC and UCEC cohort between three subtypes.
